# The potential risk of exposure to *Borrelia garinii*, *Anaplasma phagocytophilum* and *Babesia microti* in the Wolinski National Park (north-western Poland)

**DOI:** 10.1038/s41598-021-84263-0

**Published:** 2021-03-01

**Authors:** Marek Asman, Joanna Witecka, Jan Korbecki, Krzysztof Solarz

**Affiliations:** 1 Department of Parasitology, Faculty of Pharmaceutical Sciences in Sosnowiec, Medical University of Silesia, Katowice, Poland; 2 Department of Histology and Embryology, Department of Human Morphology and Embryology, Wroclaw Medical University, Wroclaw, Poland

**Keywords:** Disease prevention, Epidemiology, Ecological epidemiology, Parasitology, Pathogens

## Abstract

*Ixodes ricinus* (Acari: Ixodida) is the main vector in Europe of *Borrelia burgdorferi* sensu lato, *Anaplasma phagocytophilum* and *Babesia microti*. Wolinski National Park (WNP) is situated by the Baltic Sea and is frequently visited by tourists. The aim of the study was to determine the potential risk of exposure to tick borne infection with *B. burgdorferi* s.l., *A. phagocytophilum* and *B. microti* on the areas of WNP. In total, 394 *I. ricinus* were tested. The pathogens in ticks were detected by PCR, nested PCR, RFLP and sequencing. Altogether, pathogens were detected in 12.69% of the studied ticks. *B. burgdorferi* s.l., was shown in 0.25% of the studied *I. ricinus,* while *A. phagocytophilum* and *B. microti* were detected in 1.01% and 10.65% of studied ticks, respectively. Co-infection by *A. phagocytophilum* and *B. microti* was shown in only one *I. ricinus* nymph. Analysis of *B. burgdorferi* s.l., genospecies showed that 0.25% of the studied ticks were infected with *Borrelia garinii*. The obtained results show the potentially high human risk of exposure to tick-borne infection with *B. microti,* and the low potential risk of infection with *B. garinii* and *A. phagocytophilum* on the studied areas of WNP.

## Introduction

It is commonly known that in Europe, including Poland, *Ixodes ricinus* is a vector and/or reservoir of many pathogens including *Borrelia burgdorferi* sensu lato, *Anaplasma phagocytophilum* and *Babesia microti*. These pathogens are etiological agents of dangerous tick borne diseases such as: Lyme borreliosis, anaplasmosis and human babesiosis^1^. Lyme borreliosis is a multisystemic disease caused by the spirochete *B. burgdorferi* s.l. Three stages may be distinguished in the development of this disease which are connected with the various clinical symptoms in humans^2^. Such genospecies as *Borrelia afzelii*, *Borrelia garinii*, *Borrelia burgdorferi* sensu stricto, *Borrelia lusitaniae*, *Borrelia valaisiana*, *Borrelia spielmanii*, *Borrelia finlandensis*, *Borrelia bavariensis* are considered as pathogenic to humans and are also responsible for causing the specific clinical symptoms of this disease^3^. This is particularly true for *B. afzelii*, *B. garinii* and *B. burgdorferi* s.s. These genospecies cause acrodermatitis chronica atrophicans (ACA), neuroborreliosis and chronic arthritis, respectively^2^. In turn, *A. phagocytophilum* cause human granulocytic ehrlichiosis (granulocytic anaplasmosis). Most cases of infection by this rickettsia manifest as flu-like symptoms, conjunctivitis and lymphadenopathy^4^, whereas *B. miroti* in humans causes babesiosis. The main symptoms of this disease are flu-like symptoms similar to those in anaplasmosis. Moreover, in extreme cases, infection with this parasite in humans may affect the kidneys, lungs, myocardium, spleen and liver^5^. The aim of the study was to determine the potential risk of human exposure to tick borne infection with *B. burgdorferi* s.l., *A. phagocytophilum* and *B. microti* on the areas of the Wolinski National Park (WNP).

## Materials and method

**Figure 1:**
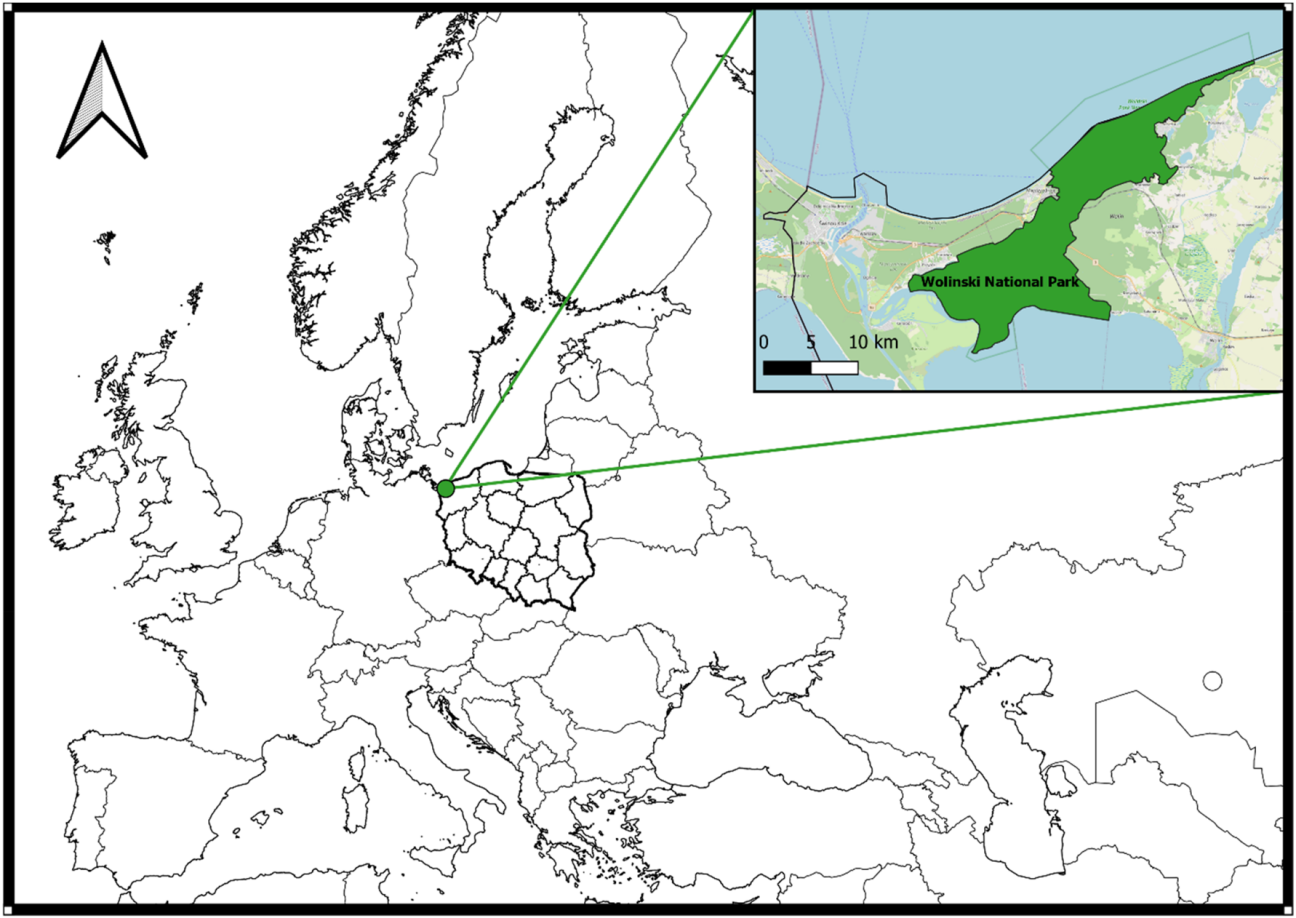
The location of the Wolinski National Park in Europe. (QGIS 3.10; https://qgis.org/pl/site/forusers/download.html).

WNP is situated by the Baltic Sea in the mid-western part of Wolin Island 53^o^57′15″N and 14^o^29′20″E in Poland (Fig. 1). In the forests covering the area of WNP pine is the dominant species, while other tree species—beech, oak and a few other tree species—occur there with a lower percentage. WNP also has a rich and varied fauna. On the area of WNP occur many species of arthropods and birds, including rare species. The forest areas of this National Park, among others, are the numerous habitats of wild boars (*Sus scrofa*), deer (*Cervus elaphus*) and roe deer (*Capreolus capreolus*), foxes (*Vulpes vulpes*), martens (*Martes martes*) and badgers (*Meles meles*). A *Bison bonasus* demonstration farm is located on the area of WNP, and nearby there are many places which are popular summer holiday destinations for visitors, mainly from Poland and Europe, but also increasingly from other countries worldwide. Humans who spend their free time in or who are working on the areas of WNP can be exposed to infestation by ticks, and potential infection with one or more tick borne pathogen.

**Figure 2:**
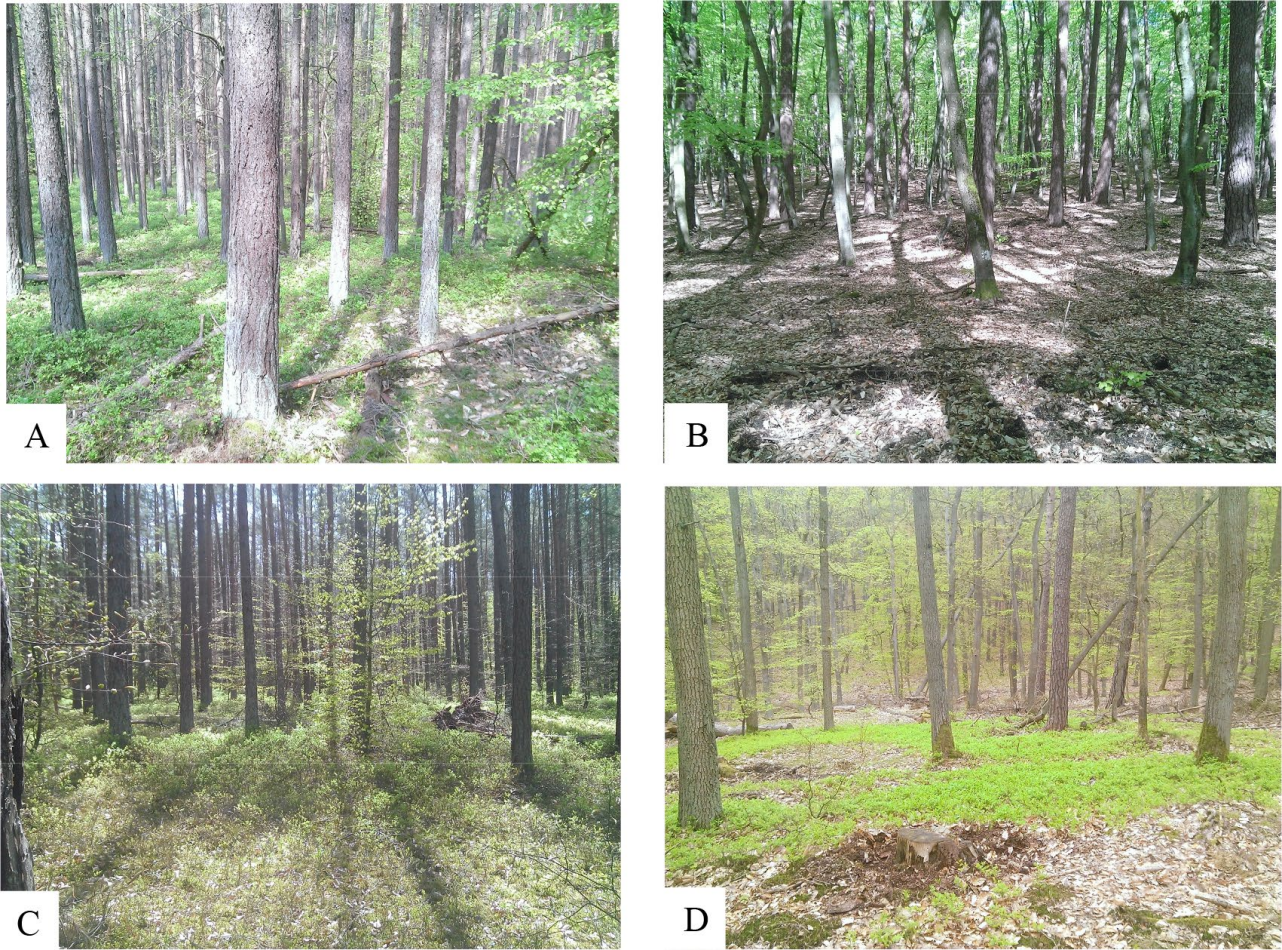
The sites sampled for ticks on the areas of Wolinski National Park. Explanation: A—Area 1; B—Area 2; C—Area 3; D—Area 4.

Ticks were collected from vegetation by the flagging method in four areas of the WNP: Area 1—53°55′53.8″N 14°28′14.6″E, Area 2—53°56′04.0″N 14°31′59.3″E, Area 3—53°55′05.5″N 14°29′03.4″E and Area 4—53°54′48.3″N 14°26′37.3″E (Fig. 2)^6^. In Areas 1 and 3, pine dominate while in Areas 2 and 4, beech and oak are the dominant species (Fig. 1). Ticks were identified to the species and developmental stage with the use of key by Nowak-Chmura^1^. DNA was isolated from 394 *I. ricinus*—15 females, 13 males, 266 nymphs and 100 larvae by the ammonia method^7^. The DNA was isolated from single adults and nymphs, while larvae were pooled in 10 individuals. Next, the concentration was measured in nanospectrophotometer PEARL (Implen, Germany) at 260/280 wave length. The samples were frozen at − 20 °C and stored for further analysis. The pathogens in ticks were detected by PCR and nested PCR. For preliminary screening for the presence of *B. burgdorferi* s.l., a pair of primers specific to the flagellin gene fragment was used^8^. In order to determine *B. burgdorferi* s.l. for the genospecies, the nested PCR–RFLP method was used. Next, *B. burgdorferi* s.l., positive samples were amplified with the use of two pairs of primers specific to the flagellin gene^9^. The nested PCR products were then cut with *HpyF3I* restriction enzyme^10^. In turn, to detect *A. phagocytophilum* and *B. microti,* the two pairs of primers specific to the 16S rRNA and 18S rRNA gene were used, respectively^11,12^. The amplicons were separated electrophoretically in 2% ethidium bromide stained agarose gels, whereas, the nested PCR–RFLP products were separated in 3.5% ethidium bromide stained agarose gels. Next, the samples were visualized under ultra violet light and photographed using an Omega 10 device (UltraLum, USA). The results were analyzed in the Total Lab computer programme (Total Lab, UK). The nested PCR product of *B. burgdorferi* s.l., was isolated from the gel by an Agarose DNA purification kit (EURx, Poland), according to the manufacture’s protocol and sequenced (Genomed, Poland). Statistical analysis was performed using CSS-Statistica for Windows 10. Statistical significance was declared at the *p* value of less than 0.05. Results were analyzed using Yates-corrected chi-square test (χ^2^).

## Results

In total, pathogens were detected in 12.69% of the studied ticks and showed 47 mono-infections and three co-infections. *Borrelia garinii* was detected in 0.25% of the studied ticks. In turn, *A. phagocytophilum* and *B. microti* were shown in 1.01% and 10.65% of the studied *I. ricinus*, respectively. Co-infection of *A. phagocytophilum* and *B. microti* was shown in only 0.76% of the studied individuals (Table 1). It should be stressed that the difference between the frequency of ticks infected with *B. microti* and *A. phagocytophilum* was statistically significant (Yates-corrected χ^2^ = 11.48; *p* = 0.0007).

**Table 1:** Number and percentage of *Ixodes ricinus* infected with *Borrelia garinii*, *Anaplasma phagocytophilum* and *Babesia microti* in the studied areas of the Wolinski National Park.

The collecting site	Number of studied ticks	1 pathogen			2 pathogens
		*Borrelia garinii*	*Anaplasma phagocytophilum*	*Babesia microti*	*Anaplasma phagocytophilum* + *Babesia microti*
Area 1	42	0 (0.00%)	2 (4.76%)	1 (2.38%)	0 (0.00%)
Area 2	90	0 (0.00%)	0 (0.00%)	0 (0.00%)	0 (0.00%)
Area 3	47	1 (2.12%)	2 (4.25%)	3 (6.38%)	0 (0.00%)
Area 4	215	0 (0.00%)	0 (0.00%)	38 (17.67%)	3 (1.39%)
Total	394	1 (0.25%)	4 (1.01%)	42 (10.65%)	3 (0.76%)

*Borrelia garinii*, was found in only one *I. ricinus* nymph collected in Area 3 (Tables 1 and 2), whereas, *A. phagocytophilum* was found in ticks collected in Areas 1 and 3. The *I. ricinus* infection level of this rickettsia in these two studied areas was similar—4.76% and 4.25%, respectively (Table 1). In turn, *B. microti* was detected in ticks collected in Areas 1, 3 and 4. The highest percentage of *I. ricinus* infected with this protozoan was showed in Area 4, while in the two other studied areas the percentage of ticks infected with *B. microti* was 2.38% and 6.38%, respectively (Table 1). This difference was statistically insignificant (Yates-corrected χ^2^ = 1.17; *p* = 0.2790).

**Table 2:** Number and percentage of *Ixodes ricinus* developmental stages infected with *Borrelia garinii*, *Anaplasma phagocytophilum* and *Babesia microti* in the studied areas of the Wolinski National Park.

Developmental stage	Number of studied ticks	1 pathogen			2 pathogens
		*Borrelia garinii*	*Anaplasma phagocytophilum*	*Babesia microti*	*Anaplasma phagocytophilum* + *Babesia microti*
Female	15	0 (0.00%)	0 (0.00%)	1 (6.66%)	0 (0.00%)
Male	13	0 (0.00%)	1 (7.69%)	0 (0.00%)	0 (0.00%)
Nymph	266	1 (0.37%)	3 (1.12%)	41 (15.41%)	3 (1.12%)
Larva	100	0 (0.00%)	0 (0.00%)	0 (0.00%)	0 (0.00%)
Total	394	1 (0.25%)	4 (1.01%)	42 (10.65%)	3 (0.76%)

Co-infection of *A. phagocytophilum* and *B. microti* was detected only in three nymphs collected in Area 4 (Tables 1 and 2), whereas in the three other studied areas co-infections with the studied pathogens were not found in the ticks.

The highest infection level of the studied tick borne pathogens was shown in *I. ricinus* nymphs, and in this developmental stage all three pathogens were detected. *Babesia microti* was shown in 15.41% of nymphs, while *B. garinii*, and *A. phagocytophilum* were detected in 0.25% and 1.12%, respectively. It should be stressed that all these differences were statistically significant (Yates-corrected χ^2^ = 14.13 and 11.48, *p* = 0.0002 and *p* = 0.0007, respectively; *p* < 0.001). Moreover, in 1.12% of studied nymphs the co-existence of *A. phagocytophilum* and *B. microti* was shown (Table 2). In turn, in the studied adults, among the *I. ricinus* ticks collected in the areas of WNP the presence of *A. phagocytophilum* and *B. microti* was shown in only one male and one female, respectively (Table 2). In turn, the presence of the studied pathogens were not found in the tested *I. ricinus* larvae. The differences in frequency of ticks infected with *B. garinii, A. phagocytophilum* or co-infected with *A. phagocytophilum* and *B. microti* between particular sites were statistically non-significant (Yates- corrected χ^2^ = 11.48; *p* > 0.05, in all cases). Ticks collected in all three areas were significantly less frequently infected with *B. microti* than in Area 4 (Yates-corrected χ^2^ = 12.5, 17.64 and 5.73; *p* = 0.0004; *p* ≤ 0.00001 and *p* = 0.0167, respectively). The difference in the prevalence of ticks infected with this protozoan between Ars 2 and 3 was also statistically significant (Yates-corrected χ^2^ = 4.30; *p* = 0.0382), whereas the remaining differences between sampling locations were statistically no-significant (Yates-corrected χ^2^ = 0.51; *p* = 0.4773).

Males were significantly more frequently infected with *A. phagocytophilum* than nymphs (Yates-corrected χ^2^ = 4.19; *p* = 0.041) or females and larvae (Yates-corrected χ^2^ = 6.38; *p* = 0.0115, in both cases) (Table 2). On the other hand, males and larvae were significantly less frequently infected with *B. microti* than nymphs (Yates-corrected χ^2^ = 14.13; *p* = 0.0002) and females (Yates- corrected χ^2^ = 5.33; *p* = 0.0210) (Table 2), whereas the difference in frequency of infected ticks between nymphs and females was statistically not significant (Yates-corrected χ^2^ = 2.50; *p* = 0.1137).

## Discussion

Generally, in Poland and in the rest of Europe, the dominant genospecies of *B. burgdorferi* s.l. is *B. afzelii*^13^. A study conducted in the areas of western Poland showed the possibility of the existence of seven genospecies of this spirochete in *I. ricinus*: *B. afzelii*, *B. burgdorferi* s.s., *B. valaisiana*, *B. bissettii*, *B. lusitaniae*, *B. miyamotoi*, *B. bavariensis*^10,14^, while a study conducted on the areas of south-western Poland showed the presence in this tick species such genospecies as: *B. afzelii*, *B. garinii*, *B. burgdorferi* s.s. and *B. valaisiana*^15^. *B. miyamotoi* was also noted in these areas^15^. On the other hand, a study conducted in areas of the Silesian Province showed only *B. afzelii*, *B. garinii* and *B. burgdorferi* s.s. in ticks^3,16,17^. The percentage of ticks infected with *B. garinii* in Poland is varied and ranged from 1.3% in north-western Poland to 22.9% in the south^3,18^. In turn, a study conducted by Stańczak et al.^12^ in Polish woodlands showed this genospecies in 14.4% of studied *I. ricinus* ticks. The results presented in this work are significantly lower that those obtained in northern and southern Poland. These differences in the tick infection level may be caused, among others, by the type of biotope which may influence the level of infection of this ectoparasites by this spirochete^19^. Moreover, the variable prevalence may suggest that various *B. burgdorferi* s.l., genospecies have different competence towards reservoir, e.g. *B. afzelii* is associated more with forest rodents than *B. garinii* and *B. valaisiana* which are more associated with birds^20–23^. However, *B. garinii* is very heterogeneous and some strains may infect ticks via rodents^24^. The lower number of ticks infected with *B. garinii* than in other regions in Poland, and lack of other genospecies related with rodents in the studied areas, may be related with an insufficient number of reservoir animals and/or their uneven distribution in WNP. Furthermore, large numbers of the host species (some species of lizards and birds) may be present in this area which may suppress the effective function of vector in spreading this spirochete in the environment. In the ecology of this spirochete, a significant role is also played by red deer (*Cervus elaphus*). Studies conducted by Wodecka^25^ confirmed the inability to survive of *B. burgdorferi* s.l. in *I. ricinus* feeding on red deer blood. The presence of the red deer population in the studied areas may also be one of the reasons for the level of tick infection with this spirochete. As WNP is an island, it can be treated as an isolated area, a fact that could also have a potential effect on limiting the migration of rodents, the main reservoirs of this spirochete.

Human granulocytic ehrlichiosis (HGE) is caused by an *Ehrlichia* species closely related to obligate intracellular bacteria which cause granulocytic in sheep, cattle, and horses^26,27^. *Anaplasma phagocytophilum* occurs mainly in ruminants, but probably also in small mammals. In turn, *E. equi*, *E. canis* and *E. chaffeensis* occur in horses, dogs and *Cervids*, respectively^28,29^. The percentage of ticks infected with *A. phagocytophilum* in Europe ranges from 0.4–66.7%^4^, whereas in Poland the percentage of *I. ricinus* infected with *A. phagocytophilum* varies up to 2.6% in south-western Poland to even 76.7% in the some forest areas of the southern part of this country^30,31^. A study conducted in the various seaside areas of northern Poland showed that the number of *I. ricinus* ticks infected with *A. phagocytophilum* ranged from 0.9% in seaside areas of the Slowinski National Park (SNP) to 14% in the suburban and urban forests areas of the Tri-City agglomeration^32,33^. The results obtained in this study are similar to those obtained in the areas of SNP, and may confirm the low presence of this pathogen in *I. ricinus* ticks on the areas of north-western Poland. Moreover, the presence of this pathogen mainly in nymphs confirms their important role in the circulation of this rickettsia in the environment, while, the presence of *A. phagocytophilum in I. ricinus* male confirms that this pathogen has the ability of transstadial passage in the *I. ricinus* population.

Studies on the occurrence of *B. microti* in Poland showed that the percentage of *I. ricinus* infected with this protozoan in the northern areas varied by up to 2.3% in the suburban and urban forests areas of the Tri-City agglomeration, and by up to 15.2 the areas of SNP^32,33^. In turn, the percentage of ticks infected with this protozoan may be higher in other region of Poland and in some areas of southern Poland may amount to even 50%^17,34^. The percentage of ticks infected with *B. microti* on the areas of WNP is slightly lower than that showed by Asman et al.^32^ on the areas of SNP. In turn, this percentage is almost four times higher than that shown by Stańczak et al.^33^ on selected areas of Tri-City agglomeration. These results may indicate that the varied potential risk of human exposure to tick-borne infection of this protozoan in different parts of Poland.

Moreover, although the main reservoir of this pathogen are rodents, the lack of this pathogens in the studied ticks on Area 2 of the current and the domination of beech and oak trees may suggest that this pathogen occurs in a concentrated manner in the environment. The circulation of *B. microti* in the natural environment takes place mainly with the juvenile stage of the ticks (Siński, 1999). The obtained results confirm this fact. In turn, the presence of the *I. ricinus* female may suggest that it has the ability to transstadial passage in the tick population, similar to the other species—*B. divergens*.

The co-existence of *A. phagocytophilum* and *B. microti* occurs very often in ticks. This phenomenon is caused by the fact that many *B. microti* are also competent for this rickettsia and even for *B. burgdorferi* s.l.^35^. However, co-infection usually occurs in ticks in a lower percentage than mono-infection. The study by Sytykiewicz et al.^36^ in central-eastern Poland showed this co-existence in 1.8% of studied ticks and in 0.9% of studied nymphs. In turn, in the areas of northern Poland this co-existence in urban and suburban forests was shown to be 10.6%^33^. However, other studies conducted in southern and eastern Poland showed the presence of these both pathogens simultaneously in 0.6% and 1.05% of the studied ticks from these areas, respectively^8,37^. The obtained results are significantly lower than these obtained by Stańczak et al.^33^ and similar to those obtained by Asman et al.^37^ on selected areas of the Kraków-Częstochowa Upland. The results of this study confirmed the possibility of the co-existence both these pathogens in a single tick. Moreover, the demonstration of this co-existence only in nymphs may be caused both by the large number of studied individuals, as well as by the fact that the developmental stage plays a key role in both of these pathogens in the environment.

## Conclusion

The obtained results show the potentially high risk of human exposure to tick-borne infection of *B. microti* and the low risk of tick-borne infection with *B. garinii* and *A. phagocytophilum* on the selected areas of WNP. In turn, demonstration of the co-existence of *A. phagocytophilum* and *B. microti* confirmed the possibility of the occurrence of more than one pathogen in a single tick. Moreover, the demonstration of the presence of the studied pathogens mainly in *I. ricinus* nymphs confirm that this developmental stage is very dangerous from the epidemiological point of view.
